# A model for the epidemiological impact of tuberculosis policy options

**DOI:** 10.2471/BLT.26.295896

**Published:** 2026-06-01

**Authors:** Sandip Mandal, Srinath Satyanarayana, Finn McQuaid, Peter J Dodd, Nicolas A Menzies, Richard G White, Nimalan Arinaminpathy, Rein MGJ Houben, David W Dowdy, Mikaela Smit, Suvanand Sahu, Carel Pretorius

**Affiliations:** a Center for Modeling and Analysis, Avenir Health, 2510 Main Street, Glastonbury, CT 06033, United States of America (USA).; b Department of Infectious Disease Epidemiology, London School of Hygiene and Tropical Medicine, London, England.; c School of Medicine and Population Health, University of Sheffield, Sheffield, England.; d Department of Global Health and Population, Harvard T.H. Chan School of Public Health, Boston, USA.; e School of Public Health, Imperial College London, London, England.; f Department of Epidemiology, Johns Hopkins Bloomberg School of Public Health, Baltimore, USA.; g Global Fund, Geneva, Switzerland.; h Stop TB Partnership, Geneva, Switzerland.

## Abstract

**Objective:**

To develop a new tuberculosis transmission model, addressing the limitations of and building on the TB Impact Model and Estimates software tool, to enable decision-makers to assess the impact of various tuberculosis interventions and allocate resources more effectively.

**Methods:**

We designed a model incorporating diagnosis and treatment pathways across public and private sectors, stratified across age groups, drug susceptibility, human immunodeficiency virus status and vaccination status. We calibrated our model using country-specific data from 29 high-burden countries and determined calibration target indicators according to national epidemic profiles. We performed the model calibration using a Bayesian adaptive Markov chain Monte Carlo process. We compare modelled and actual data for Indonesia and Nigeria.

**Findings:**

Our model calibration results showed good agreement with historical tuberculosis data. In Indonesia, we demonstrate that comprehensive implementation of the Stop TB Partnership* Global plan to end TB* interventions, including a public–private partnership, modern diagnostics, improved treatment for drug-resistant tuberculosis and a post-exposure vaccine, could enable the country to achieve the targets of the World Health Organization (WHO) End TB Strategy by 2035. In Nigeria, implementing the *National strategic plan for tuberculosis control 2021–2026* could reduce tuberculosis incidence by 27% and mortality by 37% by 2030, even without a vaccine.

**Conclusion:**

Our model provides a robust analytical foundation from which to assess the epidemiological impact of diverse interventions, prioritize investments and guide policy. The model’s open-source design and alignment with WHO recommendations make it a valuable tool for guiding evidence-based investment.

## Résumé

**Objectif:**

Mettre au point un nouveau modèle de transmission de la tuberculose qui pallie les limites de l’outil logiciel « TB Impact Model and Estimates » tout en s’appuyant sur celui-ci, afin de permettre aux décideurs politiques d’évaluer l’impact de diverses interventions de lutte contre la tuberculose et d’allouer les ressources de manière plus efficace.

**Méthodes:**

Nous avons conçu un modèle intégrant des parcours de diagnostic et de traitement dans les secteurs public et privé et stratifié les données selon les groupes d’âge, la sensibilité aux médicaments, le statut sérologique vis-à-vis du VIH et le statut vaccinal. Nous avons calibré notre modèle à l’aide de données spécifiques à 29 pays les plus touchés par la tuberculose et avons déterminé des indicateurs cibles de calibrage en fonction de profils épidémiques nationaux. Nous avons effectué ce calibrage du modèle à l’aide d’une méthode bayésienne adaptative de Monte-Carlo par chaînes de Markov. Nous avons comparé des données modélisées et des données réelles pour l’Indonésie et le Nigeria.

**Résultats:**

Les résultats du calibrage de notre modèle mettent en évidence une bonne concordance avec les données historiques sur la tuberculose. L’Indonésie a intégralement mis en œuvre des interventions du Plan mondial pour mettre fin à la tuberculose dans le cadre du partenariat « Stop TB Partnership », notamment un partenariat public-privé, des moyens de diagnostic modernes, un traitement amélioré contre la tuberculose pharmacorésistante et un vaccin post-exposition. Nous avons démontré que ces interventions permettraient au pays d’atteindre les objectifs de la Stratégie de l’Organisation mondiale de la Santé (OMS) pour mettre fin à la tuberculose d’ici 2035. Au Nigeria, la mise en œuvre du Plan stratégique national de lutte contre la tuberculose 2021–2026 permettrait de réduire l’incidence de la tuberculose de 27% et la mortalité de 37% d’ici 2030, même en l’absence de vaccin.

**Conclusion:**

Notre modèle fournit une base analytique solide permettant d’évaluer l’impact épidémiologique de différentes interventions, de hiérarchiser les investissements et d’orienter les politiques. Sa structure open source et son respect des recommandations de l’OMS en font un outil précieux pour orienter les investissements fondés sur des données scientifiques.

## Resumen

**Objetivo:**

Desarrollar un nuevo modelo de transmisión de la tuberculosis que supere las limitaciones y aproveche los avances de la herramienta informática TB Impact Model and Estimates, con el fin de permitir a los responsables de la toma de decisiones evaluar el impacto de diversas intervenciones frente a la tuberculosis y asignar los recursos de manera más eficaz.

**Métodos:**

Se diseñó un modelo que incorpora las vías de diagnóstico y tratamiento en los sectores público y privado, estratificado por grupos de edad, sensibilidad a los medicamentos, estado serológico respecto al virus de la inmunodeficiencia humana y estado de vacunación. El modelo se calibró utilizando datos específicos de 29 países con alta carga de tuberculosis, y los indicadores objetivo de calibración se determinaron de acuerdo con los perfiles epidémicos nacionales. La calibración del modelo se llevó a cabo mediante un proceso bayesiano adaptativo de Monte Carlo con cadenas de Markov. Se comparan los datos modelizados con los datos observados en Indonesia y Nigeria.

**Resultados:**

Los resultados de la calibración mostraron una buena concordancia con los datos históricos de tuberculosis. En Indonesia, se demuestra que la implementación integral de las intervenciones incluidas en el Plan Mundial para Acabar con la TB de la Alianza Alto a la TB, entre ellas una asociación público-privada, métodos diagnósticos modernos, tratamientos mejorados para la tuberculosis resistente a los medicamentos y una vacuna posterior a la exposición, podría permitir al país alcanzar los objetivos de la Estrategia Fin de la TB de la Organización Mundial de la Salud (OMS) para 2035. En Nigeria, la aplicación del *Plan Estratégico Nacional para el Control de la Tuberculosis 2021-2026* podría reducir la incidencia de la tuberculosis en un 27% y la mortalidad en un 37% para 2030, incluso sin disponer de una vacuna.

**Conclusión:**

El modelo proporciona una base analítica sólida para evaluar el impacto epidemiológico de diversas intervenciones, establecer prioridades de inversión y orientar las políticas. Su diseño de código abierto y su alineación con las recomendaciones de la OMS lo convierten en una herramienta valiosa para orientar las inversiones basadas en la evidencia.

## ملخص

**الغرض:**

تطوير نموذج جديد لانتقال السل، يعالج أوجه القصور في أداة برامج "TB Impact Model and Estimates" والبناء عليها، لتمكين صانعي القرار من تقييم أثر التدخلات المختلفة لمكافحة السل، وتخصيص الموارد بشكل أكثر فعالية.

**الطريقة:**

قمنا بتصميم نموذج يجمع بين مسارات التشخيص والعلاج عبر القطاعين العام والخاص، مصنفًا حسب الفئات العمرية، وحساسية العقاقير، وحالة الإصابة بفيروس نقص المناعة البشرية، وحالة التطعيم. وقمنا بمعايرة هذا النموذج باستخدام بيانات خاصة بكل دولة، من 29 دولة ذات عبء مرتفع، وحددنا مؤشرات المعايرة المستهدفة وفقًا للخصائص الوبائية الوطنية. وأجرينا معايرة النموذج باستخدام عملية مونت كارلو لسلسلة ماركوف التكيفية البايزية (Bayesian). ثم قمنا بمقارنة البيانات النموذجية والبيانات الفعلية لكل من إندونيسيا ونيجيريا.

**النتائج:**

أظهرت نتائج معايرة نموذجنا توافقاً جيداً مع البيانات التاريخية المتعلقة بالسل. وفي إندونيسيا، أثبتنا أن التنفيذ الشامل لتدخلات "الخطة العالمية للقضاء على السل" (Global Plan to End TB) التابعة "لشراكة وقف السل" (Stop TB Partnership)، بما في ذلك الشراكة بين القطاعين العام والخاص، ووسائل التشخيص الحديثة، وتحسين علاج السل المقاوم للعقاقير، ولقاح ما بعد التعرض للعدوى، يمكن لكل ذلك أن يدعم الدولة في تحقيق أهداف استراتيجية منظمة الصحة العالمية (WHO) للقضاء على السل بحلول عام 2035. يمكن أن يؤدي تنفيذ *الخطة الاستراتيجية الوطنية لمكافحة السل من 2021 إلى 2026 * في نيجيريا، إلى خفض معدل الإصابة بالسل بنسبة %27، ومعدل الوفيات بنسبة %37 بحلول عام 2030، حتى دون لقاح.

**الاستنتاج:**

يوفر النموذج لدينا أساسًا تحليليًا قويًا يمكن من خلاله تقييم الأثر الوبائي للتدخلات المتنوعة، وتحديد أولويات الاستثمارات، وتوجيه السياسات. إن تصميم النموذج مفتوح المصدر، وتوافقه مع توصيات منظمة الصحة العالمية، يجعل منه أداةً ذات قيمة لتوجيه الاستثمار القائم على الأدلة.

## 摘要

**目的:**

在现有结核病影响模型与测算软件工具的基础上，弥补其现存缺陷并构建一款全新的结核病宣传模型，助力决策者评估各类结核病干预措施产生的影响，实现资源更优化配置。

**方法:**

我们设计了一个模型来整合公立与私立医疗机构全流程诊疗路径，并按照年龄分组、药物药敏情况、人类免疫缺陷病毒 (HIV) 感染状态及疫苗接种状态进行分层设置。我们采用 29 个结核病高负担国家的国别专属数据对模型进行参数校准，并结合各国流行病学特征确定模型校准目标指标。我们采用贝叶斯自适应马尔可夫链蒙特卡洛抽样法完成模型校准。我们对比了印度尼西亚与尼日利亚的模型拟合数据和真实监测数据。

**结果:**

模型校准结果显示，模拟数据与结核病历史监测数据吻合度良好。研究结果表明：在印度尼西亚，全面落实遏制结核病伙伴关系《全球终结结核病规划》所列干预措施（包括公私医疗协作模式、新型诊断技术、优化耐药结核治疗以及暴露后预防性疫苗接种），有望使该国在 2035 年达成世界卫生组织 (WHO)《终结结核战略》既定防控目标。在尼日利亚，即便不引入疫苗，通过全面落实《2021-2026 年国家结核病防治战略规划》，到 2030 年该国结核病发病率可下降 27%、死亡率下降 37%。

**结论:**

本模型为评估各类干预措施的流行病学影响、优化防控投入优先事项以及制定相关政策，提供了可靠的分析基础。该模型采用开源设计，且契合世界卫生组织 (WHO) 相关建议，是指导循证卫生投入的实用工具。

## Резюме

**Цель:**

Разработать новую модель распространения туберкулеза, устраняющую ограничения программного инструмента TB Impact Model and Estimates и основанную на его наработках, чтобы предоставить лицам, принимающим решения, возможность оценивать влияние различных противотуберкулезных мер и более эффективно распределять ресурсы.

**Методы:**

Авторы разработали модель, включающую алгоритмы диагностики и лечения в государственном и частном секторах, с распределением по возрастным группам, чувствительности к лекарственным препаратам, ВИЧ-статусу и статусу вакцинации. Модель была откалибрована на основе реальных данных из 29 стран с высоким бременем туберкулеза; целевые показатели калибровки определялись в соответствии с национальными профилями эпидемии. Калибровка модели выполнялась с использованием байесовского адаптивного метода Монте-Карло с марковскими цепями. Результаты моделирования сравнивались с фактическими данными для Индонезии и Нигерии.

**Результаты:**

Итоги калибровки модели показали высокую степень согласованности модели с историческими данными по туберкулезу. На примере Индонезии продемонстрировано, что комплексное внедрение мер, предусмотренных Глобальным планом партнерства «Остановить туберкулез» (Stop TB) по искоренению туберкулеза (включая государственно-частное партнерство, современные методы диагностики, улучшенное лечение лекарственно-устойчивого туберкулеза и вакцину для применения после контакта с инфекцией), может позволить стране достичь целевых показателей стратегии Всемирной организации здравоохранения (ВОЗ) по искоренению туберкулеза к 2035 году. В Нигерии реализация *Национального стратегического плана по борьбе с туберкулезом на 2021–2026 годы* может привести к снижению заболеваемости туберкулезом на 27% и смертности на 37% к 2030 году даже без применения вакцины.

**Вывод:**

Разработанная модель обеспечивает надежную аналитическую основу для оценки эпидемиологического воздействия различных мер, определения приоритетов финансирования и выработки политики. Благодаря открытой архитектуре и соответствию рекомендациям ВОЗ эта модель становится ценным инструментом для принятия основанных на доказательных данных решений относительно финансирования.

## Introduction

Tuberculosis remains one of the deadliest infectious diseases globally, representing a significant public health challenge despite concerted efforts to control and eliminate it. In 2015, the World Health Organization (WHO) published *The end TB strategy* with the targets of a 90% reduction in incidence and a 95% reduction in mortality by 2035 relative to 2015 levels.^1^ Progress towards these goals has been constrained by structural, financial and implementation challenges, most notably a persistent funding gap that limits the scale-up of effective tuberculosis prevention, diagnostic and treatment interventions.^2^^,^^3^ Recent reductions in United States government funding for global tuberculosis programmes have further widened this gap, particularly in high-burden regions.^4^^–^^7^ In this context of insufficient resources, prioritizing high-impact interventions and quantifying the funding required to meet specific targets are critical for informed policy and investment decisions.

Mathematical modelling is a powerful tool in this context, enabling researchers and decision-makers to assess the impact of various tuberculosis interventions and allocate resources effectively.^8^^–^^13^ Transmission models have informed global and national strategies: the Stop TB Partnership *Global plan to end TB* (2016–2020, 2018–2022, 2023–2030) and the Global Fund to Fight AIDS, Tuberculosis and Malaria investment case analysis towards the 5th to 7th replenishments^14^^–^^19^ were informed by results obtained from the TB Impact Model and Estimates (TIME) software tool.^20^ Despite its broad use, this software tool has important limitations: WHO guidelines and recommendations and Global Plan to End TB strategies cannot be clearly mapped to the intervention structures; services that are not part of the national tuberculosis care programmes, including private-sector care, are not explicitly represented; large-scale vaccination is not modelled; and uncertainty plausibility bounds are absent.

Extensive use of this software tool in global applications despite these limitations prompted technical guidance groups to call for a redesigned model to inform the Global Fund Investment Case for the 8th replenishment. In response, the TB Modelling and Analysis Consortium convened our global model advisory group, comprising representatives of WHO, the Global Plan to End TB working group, the Global Fund and other modelling experts, to review existing tools and guide model improvements.

Under this guidance, we aimed to develop a new tuberculosis transmission model to address the above limitations, while building on the methodological foundations of the TB Impact Model and Estimates software tool and maintaining alignment with models used for strategic tuberculosis planning. In this paper we describe the methods used to develop a new model, present validation results for Indonesia and Nigeria, and discuss the implications for global tuberculosis control.

## Methods

### Model construction

**Fig. 1:**
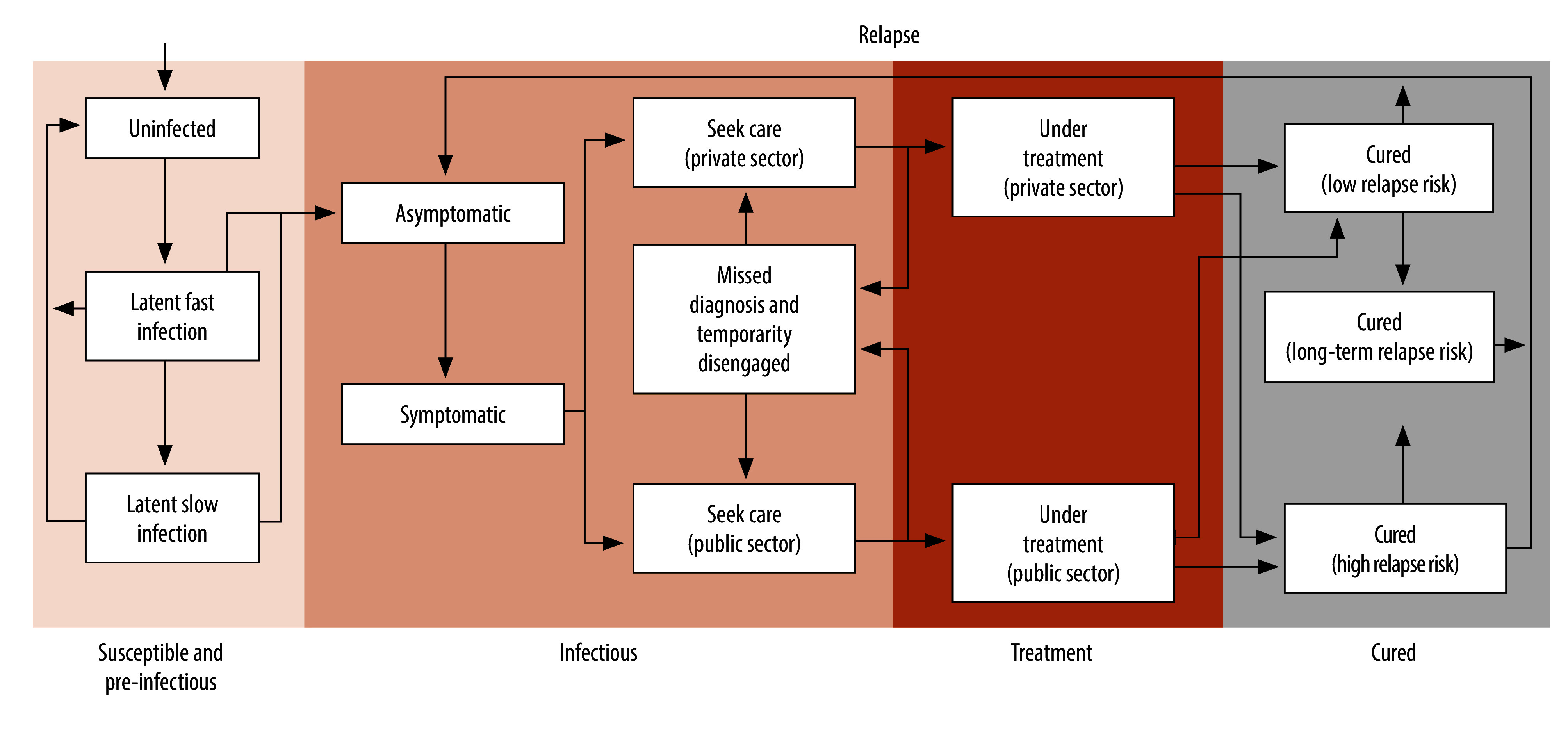
Simplified structure of newly developed tuberculosis transmission model, incorporating diagnosis and treatment pathways across public and private sectors

Note: For the full model structure is available in the online repository.^21^

We depict the simplified tuberculosis natural history model in Fig. 1, incorporating diagnosis and treatment pathways across public (national tuberculosis control programme) and private sectors. For clarity, we omit several model parameters (e.g. self-cure, exogenous reinfection and background mortality) from the diagram. For full details of model parameters and equations, please see the online repository.^21^

According to the WHO definitions updated in 2024,^22^ tuberculosis is classified as either symptomatic (individuals with typical symptoms who often seek care) or asymptomatic or subclinical (microbiologically or clinically diagnosed tuberculosis without reported symptoms during screening). Asymptomatic individuals are assumed to be less infectious.^23^^,^^24^ Symptomatic individuals seek care in the public or private sector, where successful diagnosis leads to treatment initiation; otherwise, they enter missed diagnosis or temporarily disengaged states, with potential re-entry into care unless they self-cure or die.

Our model considers that post-treatment outcomes are dependent upon the completion of treatment. We consider that patients who have completed treatment are cured and have a low relapse risk, and that individuals who are lost to follow-up during treatment enter a state of high risk of relapse. We consider those failing to complete treatment as infectious. Our model allows for the risk of occurrence of relapses within the first 2 years following recovery; we assume a stabilized lifetime relapse risk for patients who do not experience a relapse during that period.^25^^–^^27^ Our model also accounts for reinfection, with reduced susceptibility across those groups of patients with a history of infectiousness. We note that most deaths related to tuberculosis occur before the initiation of treatment, particularly in settings of low case detection.

Our model is stratified by age (0–4, 5–9, 10–14, 15–64 and ≥ 65 years, with an explicit age-contact matrix to capture age-dependent transmission); drug susceptibility (drug sensitive or rifampicin resistant); human immunodeficiency virus (HIV) status (HIV negative, HIV positive but not on antiretroviral therapy; ART; or HIV positive on ART); and vaccination status (unvaccinated, vaccinated or waning immunity). We incorporate HIV status without explicitly modelling HIV transmission dynamics.

Although our full model consists of 585 compartments (the 13 compartments depicted in Fig. 1, stratified across five age groups, two drug susceptibility categories, three HIV status groups and three vaccination status groups), we did not consider differences in tuberculosis burden between sexes, the distinction between pulmonary and extrapulmonary tuberculosis, or the effect of co-morbidities, such as diabetes. We assumed an average level of infectiousness across different forms of tuberculosis.

### Model calibration 

We calibrated our model using country-specific data from 29 high-burden countries eligible for support from the Global Fund, who together represent 90% of the tuberculosis burden in the Global Fund portfolio (see online repository for a list of these countries).^21^ We determined 13 different calibration target indicators according to national epidemic profiles, including mortality rates among HIV-negative individuals (2000 and 2022) and among people living with HIV (2022); incidence of drug-resistant tuberculosis (2022) and second-line treatment initiation (2022); overall tuberculosis incidence (2000 and 2022) and incidence among people living with HIV (2022); case notification rates (2022) and cumulative notifications for 2000–2022; as well as the proportion of symptomatic individuals among prevalent cases (2022), HIV prevalence in the general population (2022), and antiretroviral therapy coverage among people living with HIV (2022). We excluded rifampicin-resistant tuberculosis or HIV indicators where burdens were low, and we included additional data such as prevalence estimates with minimal code changes. Our data sources were a tuberculosis prevalence survey,^28^ the WHO global tuberculosis report^29^ for incidence and mortality data, and a Joint United Nations Programme on HIV/AIDS report^30^ for HIV prevalence. We used AIDS Impact Model version 6.3 (Avenir Health, Glastonbury, Untied States of America) to calculate the proportion of people living with HIV on ART. We applied a 10% uncertainty range to reported data for tuberculosis notification, cumulative notification and second-line treatment initiation to account for potential under- or overreporting in the programmatic data. 

We conducted the model calibration using a Bayesian adaptive Markov chain Monte Carlo process, which performed at least 50 000 iterations (details available in the online repository).^21^^,^^31^ We summarize our calibration data using a published reporting framework proposed (online repository).^21^^,^^32^

### Illustrative scenarios

Our model focuses on three key domains of tuberculosis intervention: prevention, diagnosis and treatment, and the model evaluates a broad range of strategies across these domains. We considered two illustrative scenarios: one examining the interventions required in Indonesia to achieve the targets defined by the WHO *The end TB strategy*,^1^ and another evaluating the impact of implementing a country-specific national strategic plan in Nigeria.^33^ In Table 1 we list the interventions assessed in our model and their coverage levels in Indonesia and Nigeria (further details available in the online repository).^21^ We assumed each intervention would commence in a specified year and scale up linearly over a defined period; we can adapt such assumptions to reflect evolving global strategies and national priorities. 

**Table 1:** Interventions and corresponding coverage levels assessed for achieving the tuberculosis incidence and mortality targets of the WHO End TB Strategy in Indonesia and for alignment with the national strategic plan in Nigeria

Intervention	Brief description of modelling activity	Coverage level to meet targets	
		Indonesia^a^ (scale-up period: 5 years)	Nigeria^b^ (scale-up period: 3 years)
**Public–private partnership**	A proportion of individuals diagnosed in private facilities receive the same standard of care as in the public sector	35%	35%
**Enhanced routine tuberculosis services**			
Improved diagnosis	Probability of successful diagnosis and treatment initiation per care-seeking visit in public sector among individuals with active tuberculosis is increased	95% from the baseline of 80%	95% from the baseline of 78%;
Improved treatment completion	Lost to follow-up is reduced	10%	5%
**Interventions for drug-resistant tuberculosis**			
Expanded drug susceptibility testing	Higher proportion of patients undergo tests soon after diagnosis	50% from the current level	75% from the current level
Shorter regimens for second-line treatment	Current second-line therapies are replaced with newly developed, shorter-duration regimens	24 months at baseline to 9 months	75% from the current level
Improved second-line treatment outcomes	Success of second-line treatment is increased with a higher proportion of patients cured	Treatment success rate increased to 70% from the baseline 50%	75% from the current level
**Upstream case-finding (symptomatic tuberculosis)**	Accelerates the diagnosis of symptomatic tuberculosis, ideally before the individual’s first care-seeking attempt	Reduced delay to diagnosis by 30%	Reduced delay to diagnosis by 17% (from 6 months to 5 months)
**Detection of asymptomatic tuberculosis**	Identify and treat individuals with asymptomatic tuberculosis before they progress to active disease	Diagnosed 30% of asymptomatic tuberculosis before developing symptoms	NA
**Preventive therapy**			
Targeting household contacts and vulnerable populations	Reduction of progression and reactivation rate by uptake among household contacts and risk groups as per WHO guidelines	5% reduction of progression (online repository)^21^	Expanded to fulfil national strategic plan targeted numbers
Targeting people living with HIV	Assumed to provide 60% protection against progression to active tuberculosis among individuals with HIV	Expanded to all people living with HIV	Expanded to all people living with HIV
**New tuberculosis vaccines**	A vaccine with 60% efficacy against both infection and disease, conferring an average duration of protection of 10 years	Rolled out to the entire adult population aged > 15 years, starting from 2028	NA

HIV: human immunodeficiency virus; NA: not applicable; WHO: World Health Organization.

^a^ To meet the targets of the WHO’s *The end TB strategy*.^1^

^b^ To align with the *National strategic plan for tuberculosis control 2021–2026*.^33^

### Assessing lives saved

By calibrating our model using incidence and mortality data from 2000 and 2022, we were able to estimate the projected number of tuberculosis deaths. To estimate the number of lives saved by tuberculosis programmes, we compared projected tuberculosis deaths with counterfactual scenarios in which public-sector tuberculosis services (supported by the Global Fund) either ceased in 2000 or remained at the 2000–2003 coverage levels without scale-up.

## Results

### Calibration

**Fig. 2:**
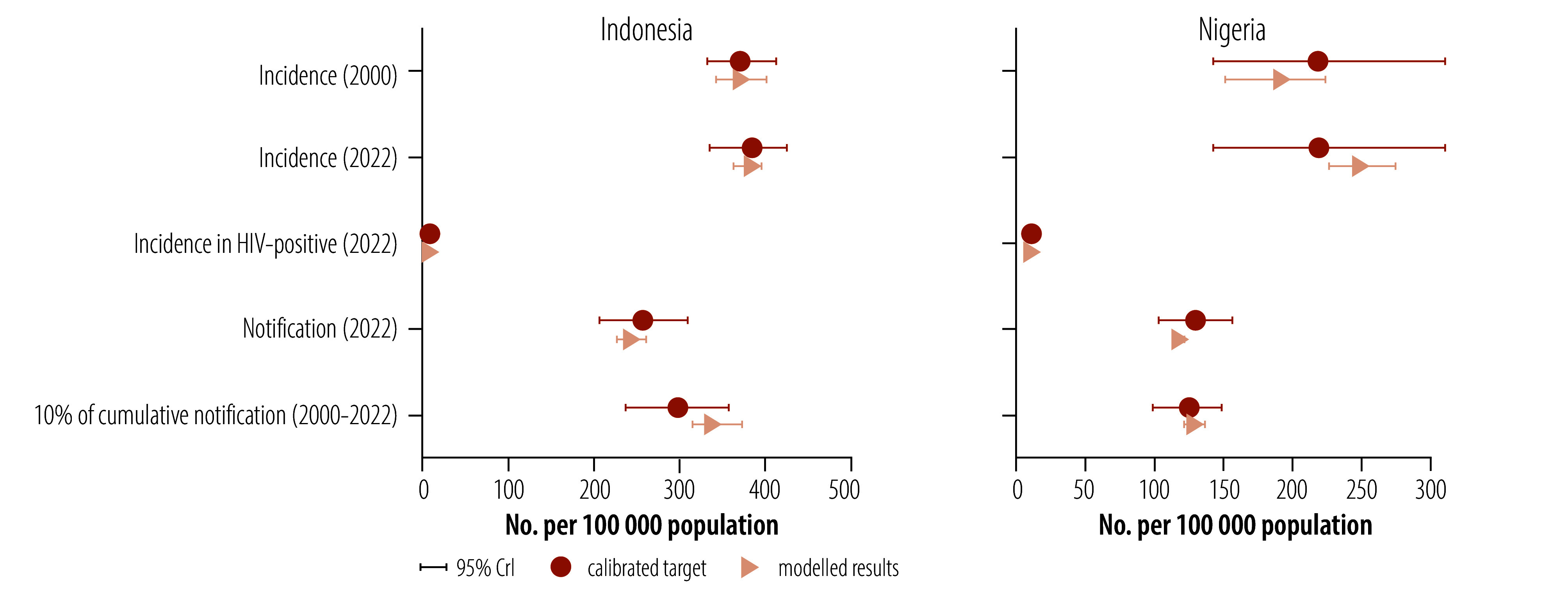
Comparison of calibration incidence with incidence estimated by a newly developed tuberculosis transmission model, Indonesia and Nigeria

CrI: credible intervals; HIV: human immunodeficiency virus.

**Fig. 3:**
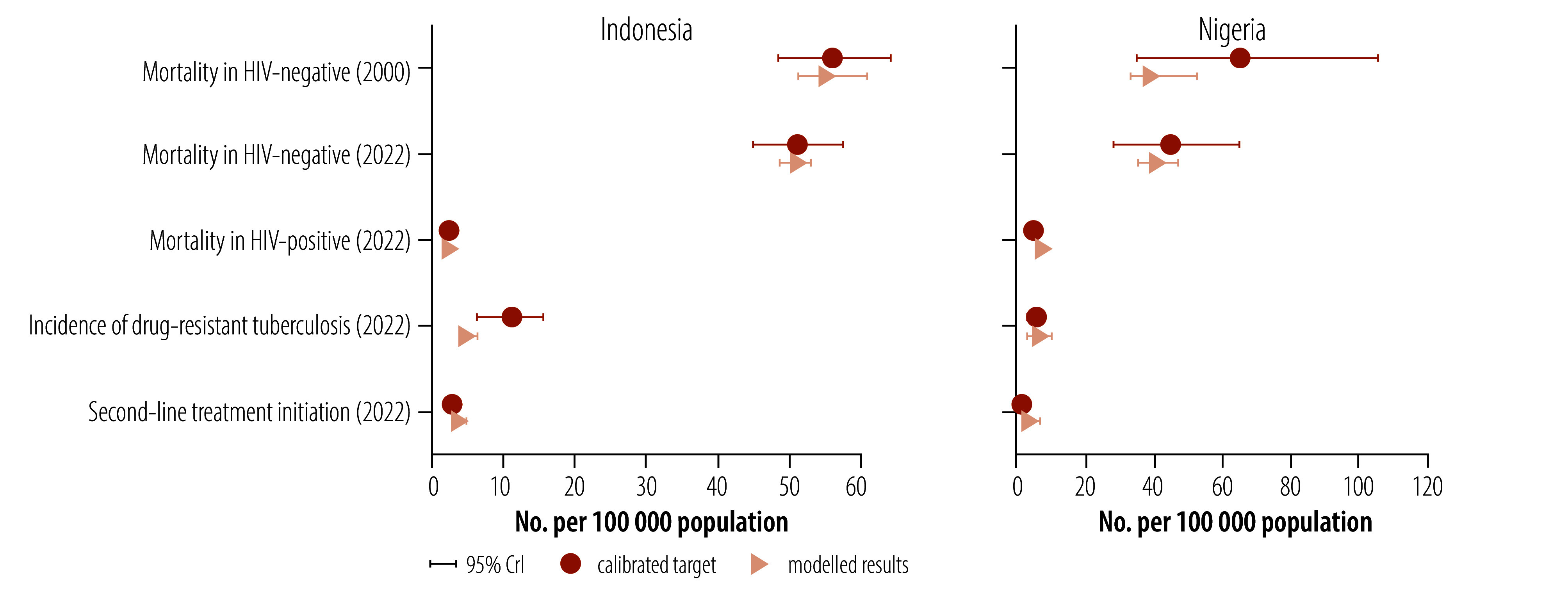
Comparison of calibration mortality with mortality estimated by a newly developed tuberculosis transmission model, Indonesia and Nigeria

CrI: credible intervals; HIV: human immunodeficiency virus.

**Fig. 4:**
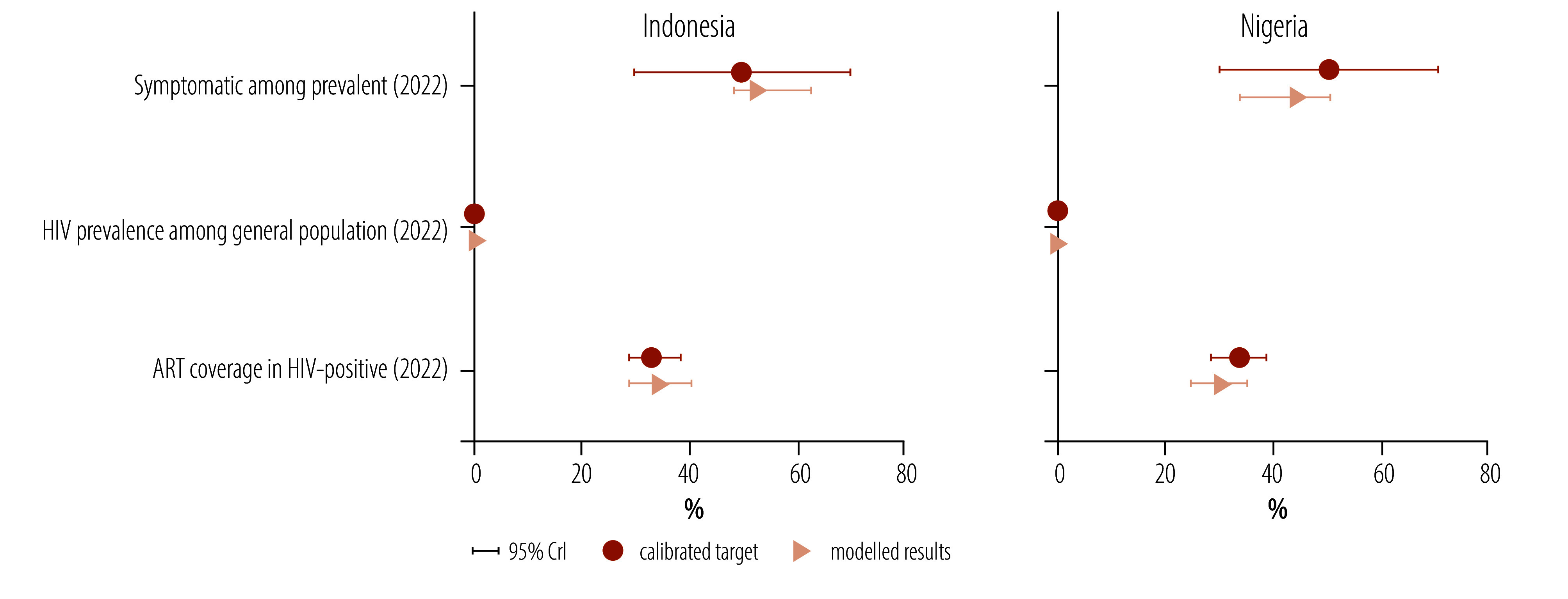
Comparison of basic calibration indicators with estimated data from newly developed tuberculosis transmission model, Indonesia and Nigeria

ART: antiretroviral therapy; CrI: credible intervals; HIV: human immunodeficiency virus.

We depict results for Indonesia and Nigeria in Fig. 2, Fig. 3 and Fig. 4, in which we compare values of calibration target indicators with modelled results for the 13 selected indicators. 

### Illustrative scenarios

#### Indonesia

**Fig. 5:**
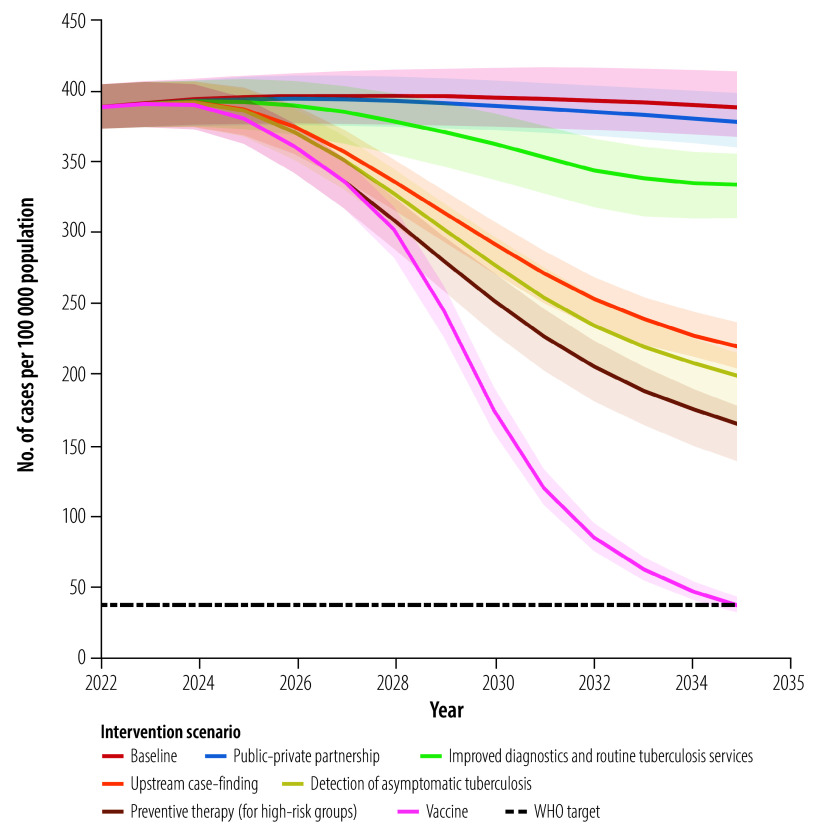
Impact of interventions on tuberculosis incidence under different intervention scenarios, Indonesia, 2022–2035

**Fig. 6:**
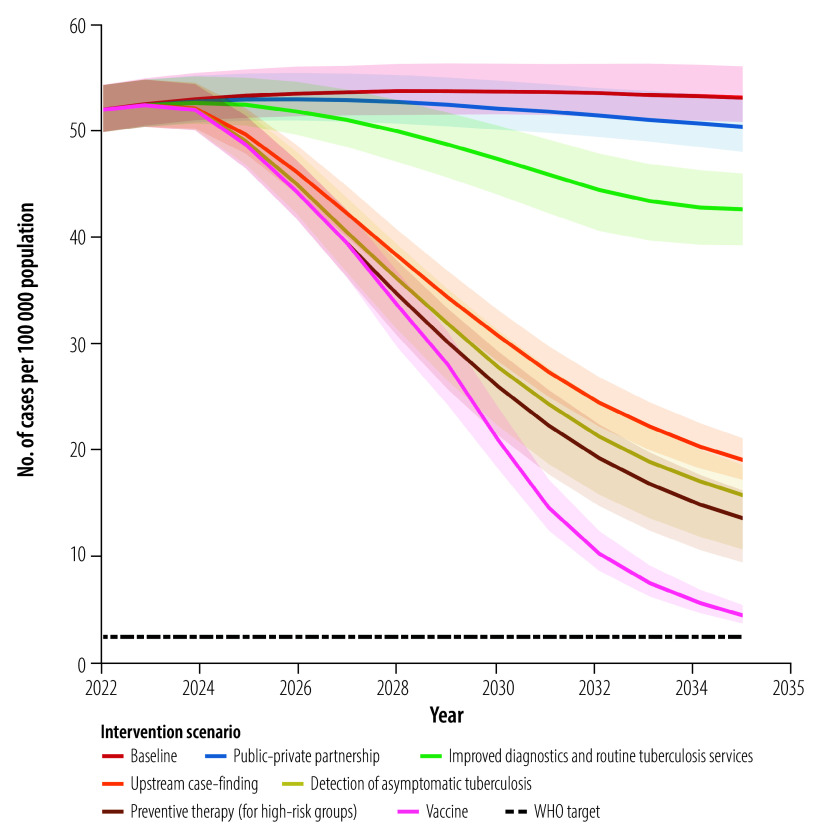
Impact of interventions on tuberculosis mortality under different intervention scenarios, Indonesia, 2022–2035

HIV: human immunodeficiency virus; WHO: World Health Organization.

Notes: intervention scenarios are outlined the Stop TB Partnership Global Plan to End TB.^14^ WHO target is from *The end TB strategy*.^1^ Shaded areas show 95% credible intervals.

Our model projects that the Indonesian health-care service could achieve the 2035 targets of *The end TB strategy* by implementing the comprehensive strategy outlined in *The global plan to end TB, 2023–2030*.^14^ Non-vaccine interventions would have to be scaled up linearly between 2024 and 2029 and maintained thereafter, and a vaccine introduced in 2028. Fig. 5 and Fig. 6 depict the projected tuberculosis incidence and mortality, respectively, under various scenarios, with current programmatic activities shown as the baseline. The credible intervals (CrIs) arise from the uncertainty in input parameters and in potential future background trends in tuberculosis burden. 

WHO: World Health Organization.

Notes: intervention scenarios are outlined the Stop TB Partnership Global Plan to End TB.^14^ WHO target is from *The end TB strategy*.^1^ Shaded areas show 95% credible intervals.

The sequential addition of interventions substantially reduces burden: for instance, implementation of a public–private partnership with improved routine tuberculosis services reduces incidence and mortality by 17% (95% CrI: 2–30) and 22% (95% CrI: 7–37), respectively, relative to 2015. Scaling all interventions except the vaccine achieves a reduction of 63% (95% CrI: 52–74) in incidence and 82% (95% CrI: 68–95) in mortality, but remains insufficient to meet the targets of the strategy. Our model shows that the introduction of a post-exposure vaccine with 60% efficacy at the population level (age, > 15 years) from 2028, rolled out over 5 years, would enable the Indonesian health-care sector to achieve the targets of the strategy.

#### Nigeria

**Fig. 7:**
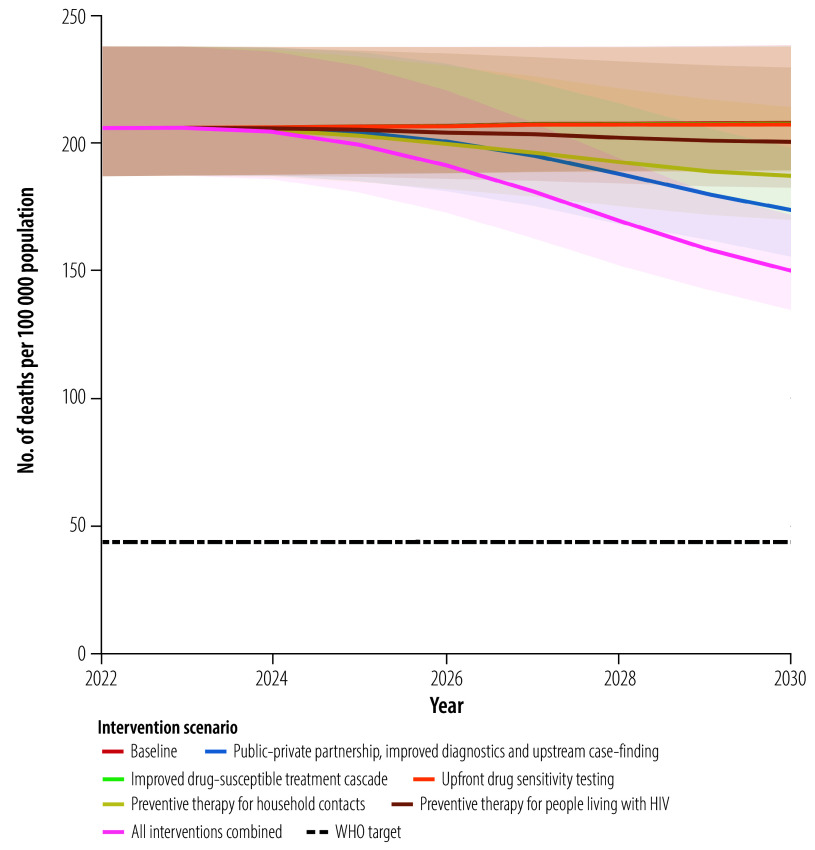
Impact of interventions on tuberculosis incidence under different intervention targets as described in the proposed National Strategic Plan, Nigeria,2022–2030

HIV: human immunodeficiency virus; WHO: World Health Organization.

Notes: intervention targets as described in the *National strategic plan for tuberculosis control*.^33^ WHO target is from *The end TB strategy*.^1^ Shaded areas show 95% credible intervals.

**Fig. 8:**
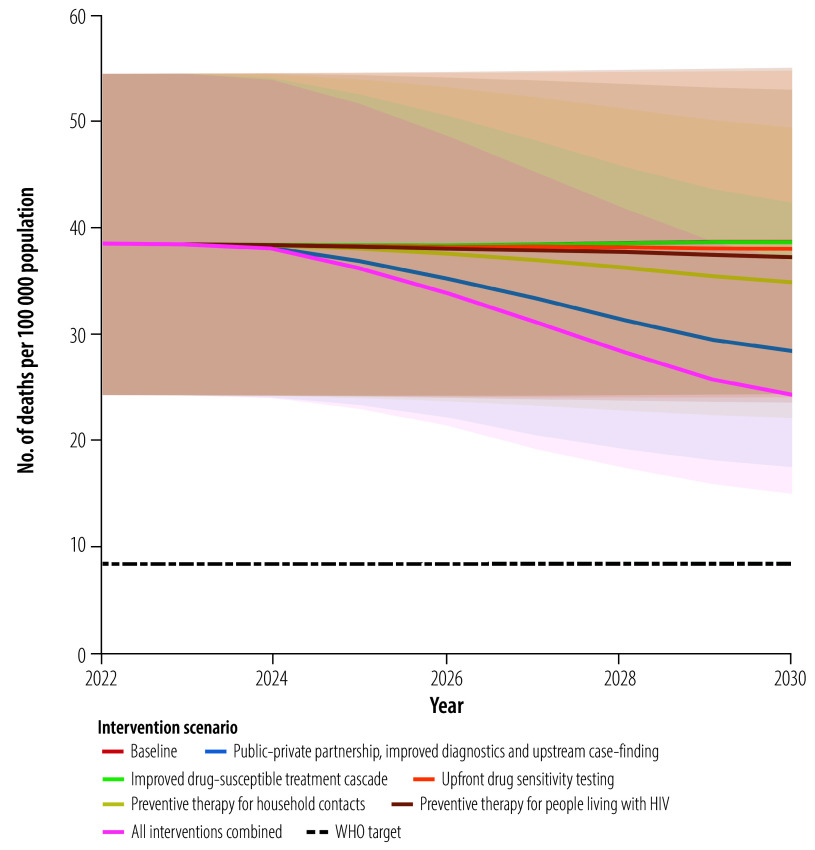
Impact of interventions on tuberculosis mortality under different intervention targets, Nigeria 2022–2030

HIV: human immunodeficiency virus; WHO: World Health Organization.

Notes: intervention targets as described in the *National strategic plan for tuberculosis control*.^33^ WHO target is from *The end TB strategy*.^1^ Shaded areas show 95% credible intervals.

We illustrate the projected impact of the full-scale implementation of Nigeria’s *National strategic plan for tuberculosis control 2021–2026* during 2022–2030 in Fig. 7 and Fig. 8 with curves representing individual interventions. The wide credibility bounds reflect the substantial uncertainty in available epidemiological data and calibration targets for Nigeria, which is appropriately propagated through the model. The greatest reduction in tuberculosis burden is achieved when all interventions are implemented concurrently. In the absence of vaccination, the national strategic plan interventions alone are estimated to reduce tuberculosis incidence by 27% (95% CrI: 14–39) and mortality by 37% (95% CrI: 11–57) by 2030, relative to 2022 levels.

### Lives saved

We used our model to estimate the returns on investment from past tuberculosis programme scale-up, starting from the first year of Global Fund investment. We performed our analysis at the country level for the 29 high-burden countries (online repository)^21^ and then aggregated our results to estimate the impact for all countries eligible for Global Fund support.

**Fig. 9:**
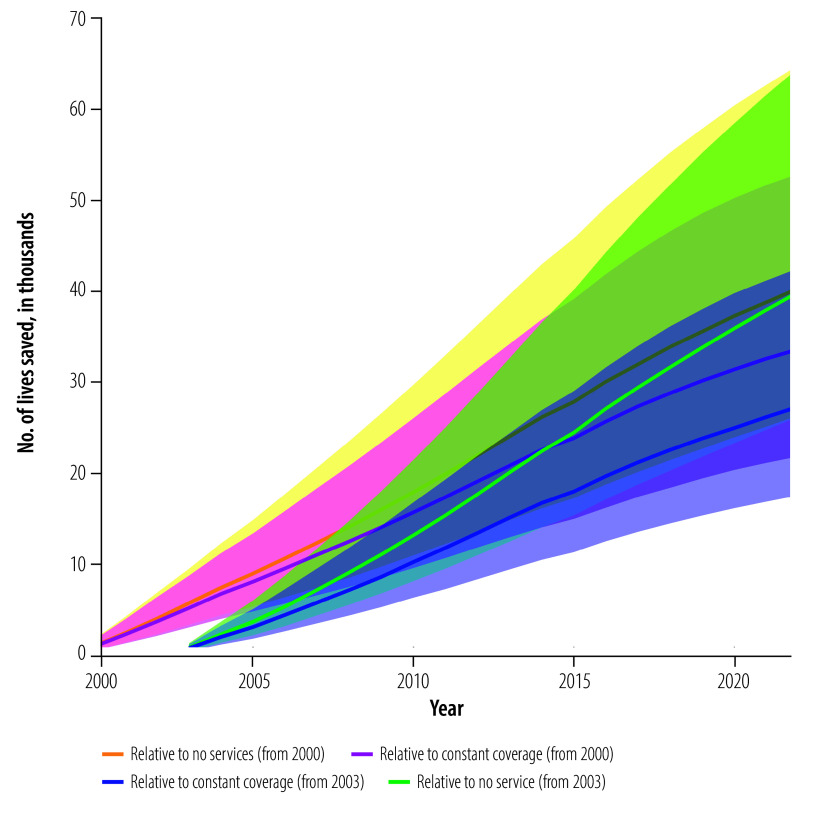
Estimated number of lives saved annually by tuberculosis programmes compared with no service and constant coverage scenarios, Indonesia

Notes: no service refers to a counterfactual scenario in which all public-sector tuberculosis services had ceased in 2000 or 2003, and constant coverage refers to a counterfactual scenario in which services had remained at their 2000 or 2003 levels without scale-up. Shaded areas show 95% credible intervals.

In Fig. 9 we illustrate country-level results for Indonesia, showing estimated annual lives saved under two scenarios. Compared with a counterfactual scenario in which all public-sector tuberculosis services had ceased in 2000 or 2003, an estimated 41 400 (95% CrI: 31 200–48 600) or 41 000 (95% CrI: 31 200–48 300) lives, respectively, were saved in 2022 as a result of Global Fund support. Similarly, compared with a scenario in which services had remained at their 2000 or 2003 levels without scale-up, estimated lives saved in 2022 were 34 100 (95% CrI: 26 300–40 700) and 27 200 (95% CrI: 21 800–33 800), respectively, as a result of Global Fund support. 

## Discussion

Our enhanced global tuberculosis transmission model addresses the key limitations of existing frameworks, and demonstrates flexibility and high accuracy in evaluating national and global tuberculosis control strategies. By capturing critical epidemiological heterogeneities, our model enables policy-relevant assessments of potential interventions across prevention, diagnostic and treatment pathways. The model’s capacity to simulate upstream case-finding and vaccine roll-out dynamics, including waning immunity and efficacy, further strengthens its utility for evaluating future strategies.

For Indonesia, our findings indicate the magnitude of coverage levels required to meet the targets of WHO’s *The end TB strategy*; however, the feasibility of achieving these targets depends on health system capacity as well as the country’s economic constraints. Our model therefore does not determine whether a country can achieve the goals, but projects results that can serve as a starting point for discussion on what would be required to meet incidence and mortality targets. In contrast, our analysis of the *National strategic plan for tuberculosis control* *2021–2026* of Nigeria illustrates that well-aligned, nationally tailored interventions can achieve substantial impact even without a vaccine. These scenario analyses underscore the importance of coherent national planning and coordinated public–private implementation, inform the Global Fund Investment Case for the 8th replenishment and allow an estimation of primary health-care utilization savings.^34^

To ensure alignment with WHO screening and tuberculosis care guidelines,^35^ as well as *The global plan to end TB, 2023–2030*, we also developed a target population component to interface between the global costing and impact models (available in the online repository).^21^ These populations are described in detail in a separate paper on the costing component that we developed alongside the transmission component.^36^ We aggregated tuberculosis prevention, diagnostic and treatment variables across these populations to inform the global transmission model, enabling cost and impact analyses aligned with WHO guidelines. These functionalities make our model a valuable tool for donor decision-making, based on investment outcomes such as those presented in the most recent Global Fund Investment Case.

Alignment of the target population component with the WHO Integrated Health Tool framework^37^ enables our model to be used within the tuberculosis module of the framework for national planning and decision-making, with user-defined inputs allowing closer alignment with country-specific contexts than for typical global analyses.

A key strength of our model lies in its open-source design and publicly accessible,^38^ enabling transparency, reproducibility and adaptability to country-specific epidemiology and programmatic structures.

However, several limitations must be acknowledged. First, although our model captures age-dependent mixing and major determinants of tuberculosis transmission such as HIV status, it excludes other important factors, as mentioned in the section Model construction above. Second, HIV is included through stratification, but dynamic modelling of HIV transmission and ART scale-up is beyond the scope of our model. Third, another limitation is our reliance on generalized assumptions where country-specific data are lacking, particularly for treatment adherence and the quality of private-sector health care. Although Bayesian calibration with wide priors addresses some uncertainty, improved local data would enhance model fidelity. Fourth, although our model is efficient for global analyses, large-scale multicountry scenario testing remains computationally demanding. Fifth, because of limited subnational data availability, our model was calibrated using national-level data from WHO global tuberculosis reports, resulting in national-average estimates that may not capture within-country heterogeneity. Where subnational data exist, the model can be recalibrated to generate localized estimates.

Although model-based analyses support strategic decision-making, they cannot replace empirical evidence. Such analyses should be complemented by implementation research. Such validation requires sustained funding, stronger country-level data systems and close collaboration with national tuberculosis programmes.

Our global tuberculosis model represents a considerable step forward in producing model-based information for strategic programme planning. Our model provides a robust analytical foundation from which to assess the epidemiological impact of diverse interventions, prioritize investments and guide policy. As funding constraints continue to shape global health priorities, such modelling tools are indispensable for developing cost-effective and evidence-based strategies.
